# A Clinical Case of Patient Carrying Rare Pathological PSEN1 Gene Mutation (L424V) Demonstrates the Phenotypic Heterogenity of Early Onset Familial AD

**DOI:** 10.3389/fpsyt.2019.00857

**Published:** 2019-12-11

**Authors:** Kaloyan R. Stoychev, Maya Stoimenova-Popova, Petranka Chumpalova, Lilia Ilieva, Mohamed Swamad, Zornitsa Kamburova-Martinova

**Affiliations:** ^1^Department of Psychiatry, Medical University Pleven, Pleven, Bulgaria; ^2^Department of Neurology, Sveti Panteleimon Hospital, Pleven, Bulgaria; ^3^Department of Health and Aging Unit, King’s College Hospital, London, United Kingdom; ^4^Department of Microbiology and Medical Genetics, Medical University Pleven, Pleven, Bulgaria

**Keywords:** phenotypic heterogeneity, whole exome sequencing, PSEN1 mutation, genetic inheritance, early onset Alzheimer’s disease (EOAD)

## Abstract

Dementia comprises several neurodegenerative disorders with similar neuropsychiatric features and Alzheimer’s disease (AD) is the most common of them. Genetic factors are strongly implicated into its etiology especially for early-onset cases (EOAD) occuring before the age of 65. About 10% of these are inherited in autosomal dominant fashion *via* pathogenic polymorphisms in three genes— APP, PSEN-1, and PSEN-2. Despite genotypic clarity, however, phenotypic variability exists with different symptom constellations observed in patients with identical mutations. Below, we present a case of a 39-year-old male with a family history for early onset dementia who was referred to our department with anamnesis for abrupt behavioral change 7 months prior to hospitalization—noticeable slowing of speech and reactivity, impaired occupational functioning and irritability, followed by aphasic symptoms and transient episodes of disorientation. He was followed up for 2 years and manifested rapidly progressing cognitive decline with further deterioration of speech, apraxia, acalculia, ataxia, and subsequently bradykinesia and tremor. Based on the clinical and neuroimaging findings (severe cortical atrophy), familial EOAD was suspected and a whole exome sequence (WES) analysis was performed. It identified a heterozygous missense variant Leu424Val (g.71074C > G) in PSEN-1 gene considered to be pathogenic, and only reported once until now in a Spanish patient in 2009. Despite genotype identity however, distinct phenotypic presentations were observed in the two affected subjects, with different neuroimaging findings, and the presence and absence of seizures in the Spanish and Bulgarian case, respectively. Besides, myoclonus and spastic paraparesis considered “typical” EOAD clinical features were absent. Age of symptom onset was consistent with two of the reported mutations affecting 424 codon of PSEN-1 gene and significantly earlier than the other two implying that factors influencing activity of PSEN-1 pathological forms are yet to be clarified. Furthermore, our patient had co-occurring lupus erythematosus (LE) and we suggest that this condition might be etiologically linked to the PSEN-1 mutation. In addition to illustrating the symptomatic heterogeneity of PSEN-1 caused EOAD, our study confirms that in patients presenting with early cognitive deterioration and family history for dementia, WES can be especially informative and should be considered as a first-line examination.

## Background

Several neurodegenerative disorders are grouped under the umbrella term dementia (1) and these include Alzheimer’s disease (AD) comprising more than 60% of cases, vascular dementia (VD), dementia with Lewy bodies (DLB), and frontotemporal dementias group (FTD) (2). Their symptoms often overlap (3) and precise diagnosis is difficult but may be boosted by several approaches, including genetic testing. (4).

Genetic background of dementia syndrome is widely recognized (5). It may present as a complex condition with many genes of small effect contributing to illness risk or, more rarely, as an autosomal dominant disease running in families (6). Such forms are almost exclusively found among early onset dementia (EOD) patients, i.e., diagnosed under the age of 65. With prevalence exceeding 40.0% of patients for some dementia subtypes (7), EOD is evidently not sporadic (8), and the genetic underpinnings of AD and FTD have been most extensively studied (9).

Early-onset AD (EOAD) represents 5.5% all AD cases (10) and in 10% of the affected, a familial model of inheritance is seen (11). Its clinical course is markedly accelerated (12) and myoclonus in the early stages may be common (13). To date, three major EOAD causative genes are recognized (14): mutations in Presenilin-1 (*PSEN-1*, 14q24.2) are identified in up to 50% of patients (15), abnormal forms of Amyloid Precursor Protein gene (*APP*, 21q21.3) are found in 15-22% (16), and Presenilin-2 (*PSEN-2*, 1q42.13) pathological variants account for less than 2.5% (17). So far, 51 pathological forms have been reported for *APP*, 220 for *PSEN-1*, and 16 for *PSEN-2* (18). All but a few of them are involved in the pathogenesis of AD through the process of formation of amyloid-β protein built plaques, which are a histopathologic hallmark of the disease and a central component of the “amyloid cascade hypothesis” for AD (19).

Several phenotypic clues point to the involvement of a particular gene. While spastic paraparesis and “cotton wool plaques” (20) are associated with a *PSEN-1* mutations, cerebral amyloid angiopathy with cerebral haemorrhages is typical for *APP* ones (21) and families with *PSEN-2* caused AD are primarily of Volga-German origin and with later onset of symptoms (22). However, recent studies show that clinical phenotype is not rigidly linked to aetiological genotype and may be modified by a number of factors such as previous experience, cognitive activity and epigenetic mechanisms (23). *PSEN-1* mutations are a good example of that given the remarkable heterogeneity in neuropsychiatric features among affected individuals whose symptoms are often nearly identical to those seen in FTD or DLB (24).

Below, we describe a clinical case of an EOAD patient carrying a rare form of *PSEN-1* mutation who was followed-up for 2 years after disease onset. His clinical presentation exemplifies the large symptomatic variability of EOAD dementia even in its aetiologically clarified monogenic forms.

## Case Presentation

The subject first presented for consultation in October 2016 (aged 39) at the Department of Psychiatry of Pleven University Hospital with a history of abrupt behavioral change 7 months prior to hospitalization. Slowing of speech and responsivity as well as impaired occupational functioning were noticed first (the patient was working as a coupier in a small casino at that time). Several months after that, mild dysarthria and difficulties finding the right words (i.e., dysnomic manifestations) occurred, followed by emotianal lability with frequent and brief episodes of sadness (crying) or irritability with verbal outbusrsts. Transient episodes of disorientation and perplexity appeared after that. Relevant medical history included head trauma without loss of consciousness in 2014 and a skin form of lupus erythematodes since 2007, currently in remission. These conditions were not considered as causally related to presenting symptoms. Premorbid functioning was appropriate to social background and academic level (high school) and what attracted attention was family history showing that the patient’s father had been diagnosed with dementia syndrome started at about 44 years of age and lead to death in a psychiatric institution 4 years after that. Unfortunately, available medical records were limited, with CT data for cortical atrophy described as an instrumental finding and rapidly progressing severe memory disturbance and dyspraxia followed by avolitional-apathy syndrome pointed out as clinical features.

**Figure 1:**
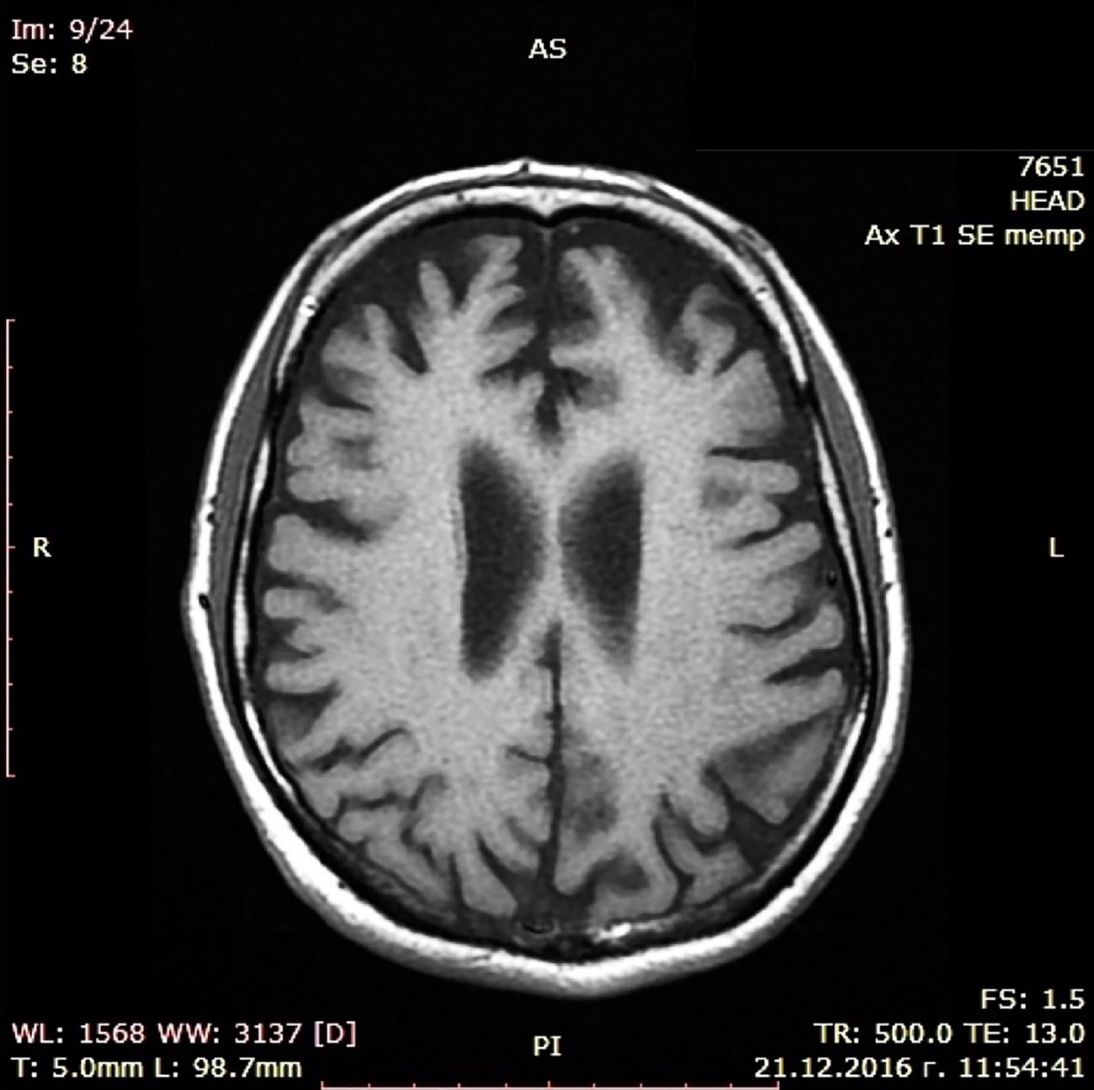
MRI scan (transversal).

**Figure 2:**
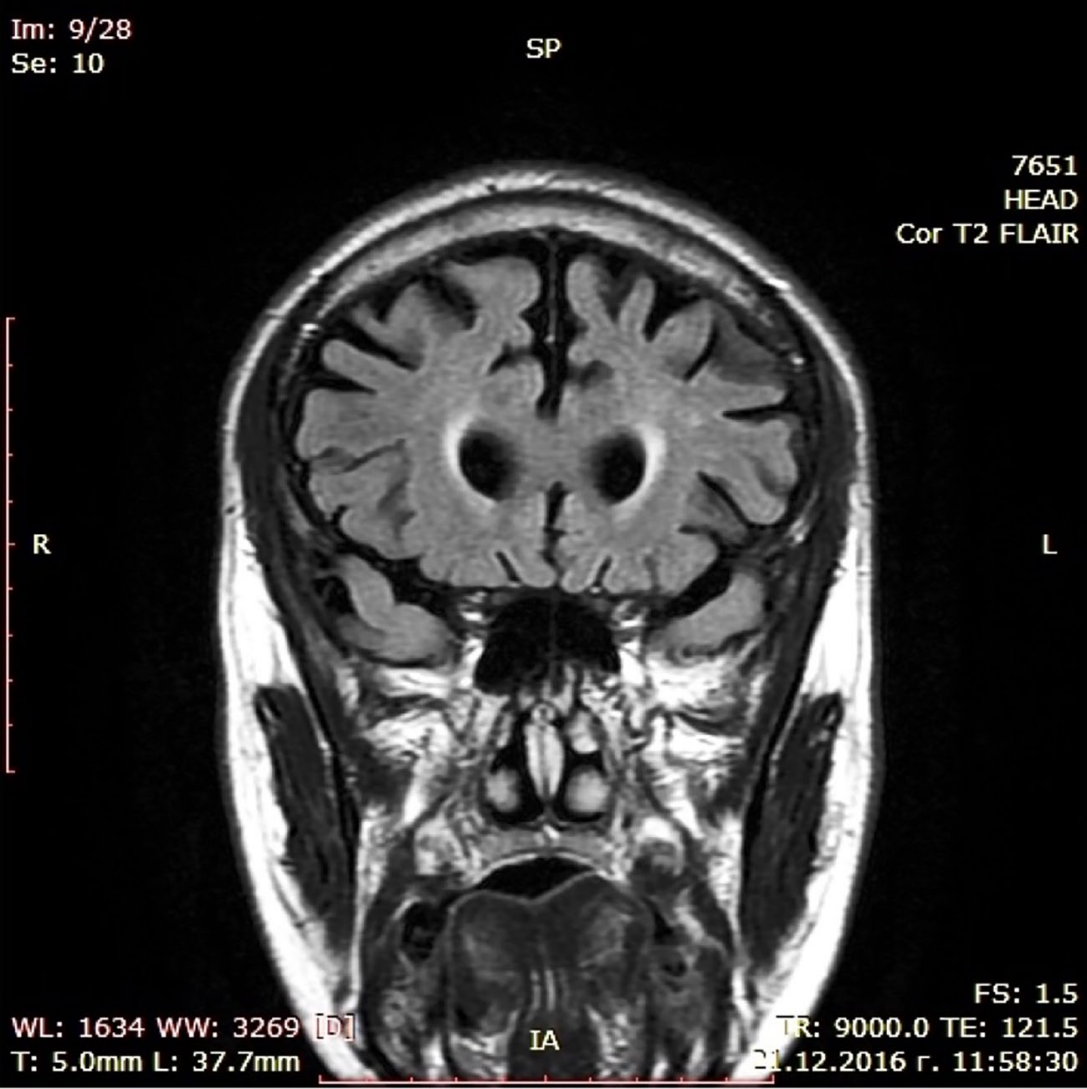
MRI scan (coronal).

During a short hospital stay, CT scan and a battery of cognitive tests were performed showing moderate cortical atrophy in par with a moderately severe dementia syndrome (MMSE score of 14) presenting with significant short-term memory impairment, problems with concentration, and sustained attention during cognitive tasks, substantially damaged abstract thinking with reduced ability to form and understand concepts, difficulties with verbalization of his own mental experiences. Constructional dypraxia was prominent together with writing problems (dysgraphia) while biographical memory was relatively spared. Laboratory check-up including CBT, renal and liver biochemistry, serum glucose, lipid profile, vitamin B12, folate and TSH levels, urine test as well as immunological examination for luetic and HIV infections did not reveal deviations. EEG examination was not judged to be indicated since patient did not show epileptic activity. Cerebrospinal fluid (CSF) biomarkers test and PET were not done because these diagnostic approaches are still with limited availability in Bulgaria. Dementia syndrome was set as a working diagnosis, treatment with combined medication containig Ω-3 fatty acids, Vitamin B12, folic acid, Vitamin E, Gingko biloba extract, and phosphatidilserine was initiated, and he was referred to a neurologist. The latter arranged an MRI scan in November 2016 confirming the CT finding of enlarged brain convexity sulci predominantly in the temporal and frontal regions (**Figures 1** and **2**).

Besides, a vertigo syndrome was noted along with tinnitus, mild horizontal nystagmus, and positive Romberg sign. Vinpocetine 10-mg TID and memantine 5-mg BID were added to treatment.

**Figure 3:**
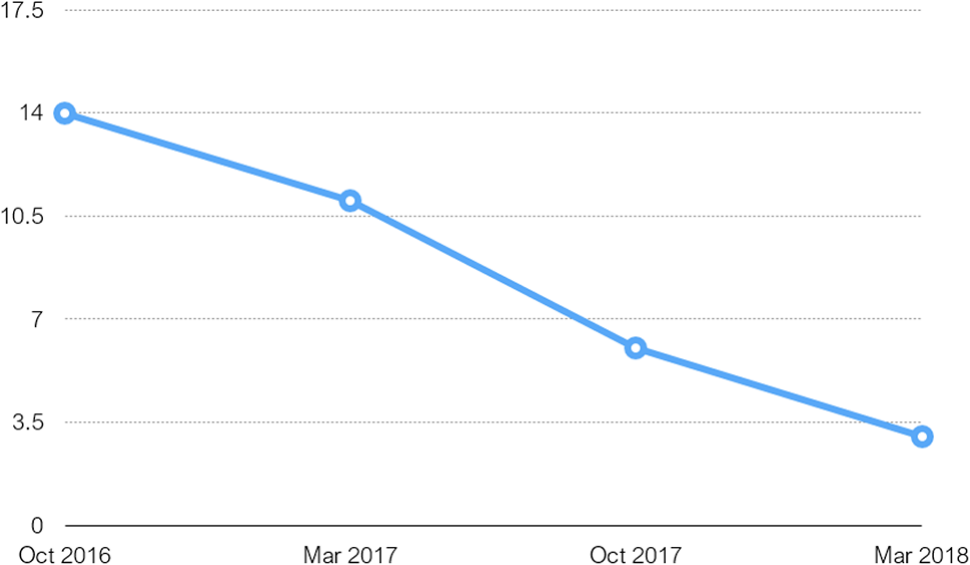
Table charting MMSE scores over period of 17 months from first test done on initial admission.

Subsequently, the subject was followed-up every 6 months and showed rapid cognitive deterioration reflected in MMSE scores—from 11 in March 2017 to 3 in March 2018 (**Figure 3**) with progressive global aphasia, acalculia, apraxia, and bradykinesia. Ataxia and gait disturbances occurred in this period and episodes of spontaneous verbal and motor excitement were reported, sometimes accompanied by verbal aggression outbursts. Mild hand tremor and parkionsonian like facial expression were noticed during the neurological examination in October 2017. In March 2018, the tremor became severe, resulting in total loss of capacity for self-care. From the summer of 2018 onward, the patient was already bed-ridden with persisting coarse hand tremor increasing in intentional movements and almost missing at rest. Both global aphasia and visual agnosia were extremely expressed.

Based on clinical presentation and family history, an early onset familial AD was set as primary diagnosis and a complete blood sample for genetic testing was obtained after consent from caregivers in November 2018. Unfortunately, subject died soon after that from complicated respiratory infection, and a pathological examination could not be performed. Whole exome sequence (WES) analysis for 21 targeted genes associated with an array of inheritable neurodegenerative disorders was executed *via* a specially designed diagnostic panel (https://blueprintgenetics.com/tests/panels/neurology/dementia-panel/). It identified a heterozygous missense variant (g.71074C > G) Leu424Val in *PSEN-1* gene and this finding, confirmed by bidirectional Sanger sequencing, was judged to be pathogenic, i.e., causing clinical symptoms. To our best knowledge, the Leu424Val mutation has been reported in literature only once until now in 2009 (25) but the course and pattern of associated neuropsychiatric symptoms were different.

## Discussion

The presented case report describes a rare pathogenic variant of *PSEN-1* gene carried by a male subject with symptoms of cognitive decline started at about 38 years of age and with a family history of early onset dementia.

The *PSEN-1* gene (14q24.2) encodes presenilin-1, which is part of γ-secretase—a transmembrane protein complex responsible for the proteolytic cleavage of amyloid precursor protein (26). Mutations in *APP*, *PSEN-1*, and *PSEN-2* genes produce the same pathogenic activity, precisely an increase in the ratio of amyloid β1_42_ amino acid isoform to β1_40_ amino acid isoform levels (27), resulting in aggregation of the peptide into oligomers and ultimately amyloid fibriles forming plaques (28). This same mechanism has been confirmed for the *PSEN-1* Leu424Val (g.71074C > G) variant in a functional study by Sun et al. from 2017 (29).

**Figure 4:**
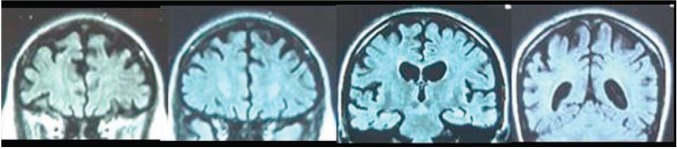
Coronal MRI slices (anterior to posterior) from a 35 years old female patient carrying L424V *PSEN-1* mutation. From A. Robles et al. (2009). American Journal of Alzheimer's disease and othe dementias, 24(1); p.41 copyright © 2019. Reprinted by Permission of SAGE Publications, Inc.

According to AD and FTD Database (http://www.molgen.ua.ac.be/ADMutation) (g.71074C > G) Leu424Val mutation has been reported previously only once in 2009 by Robles et al. in Spain (25), in a female with early onset FTD. At the age of 26, she manifested symptoms of anorexia nervosa, at the age of 30, memory and attention deficit occurred, and at 34, abnormal behavior with impulsivity, aggression, and dysexecutive disorder were present. At 36, she showed aphasia, stereotyped behavior, hyperreflexia, grasping reflex, urinary incontinence, myoclonus, and seizures. Brain CT and SPECT showed diffuse cortico-subcortical atrophy (**Figure 4**) and fronto-temporo-parietal hypoperfusion. An identical mutation has also been reported by Ryman et al. (30) in an AD patient with age of onset 26 years, but it is most likely that it refers to the abovementioned case.

Notably, four other variants affecting the same PSEN-1 codon/amino acid - Leu424Arg (g.71075T > G), Leu424His (g.71075T > A), Leu424Phe (g.71074C > T) and Leu424Pro (g.73685864T > C) - have been reported in association with early onset familial AD (31)—**Table 1**. The first one has been originally described by Kowalska et al. (32, 33) in a Polish pedigree from the region of Poznan with familial AD with age of onset of 30-35 years, fast progression and typical clinical presentation. Interestingly, 10 years later the same mutation was replicated in a 38 year old Dutch male with familial AD but with much more diverse phenotype observed in the pedigree, including spastic paraparesis, frontal executive functional impairment, gait disturbances, ataxia, epilepsy, and hallucinations (34). Finally, this genotype was confirmed in 2014 in another patient from Poland—a 36 years old woman with positive family history for early dementia and atypical presentation initially suggestive of Huntington disease and later of familial prion disease (35)—**Table 1**. The Leu424His (g.71075T > A) variant was announced in 2005 by two teams—as an atypical AD in a 39 year old female patient from Poland (36) and in three subjects from a French family with autosomal-dominant AD in whom the onset of symptoms were between 38 and 42 years of age (37). Again, identical genotype produced distinct phenotypic manifestation. Specifically, marked clinical atypism was noted in the Polish case with symptoms suggestive of FTD (social disinhibition, personality change) and DLB (visual and auditory hallucinations, stereotypic behaviors, rigid-akinetic movement disorder) observed. In 2006, the Leu424Phe (g.71074C > T) mutation was identified in a Bulgarian pedigree with later onset atypical familal AD phenotype associated with significant behavioral abnormalities and white matter changes on neuroimaging (38) and has not been replicated as yet. The last and most recent abnormal variant—Leu424Pro (g.73685864T > C) —has been described in 2019 by Guven et al. (39) in a 51 years old Turkish male proband with EOAD demonstrated by deterioration of short-term memory and visuospatial skills with insidious beginning and slow progression. For understandable reasons, no replication has been reported so far.

**Table 1:** Currently known pathological mutations at position 424 of the PSEN-1 gene with presenting phenotype in affected individuals/families.

Mutation	Proband	Symptom course	Neuroimaging/Biomarkers/Neuropathology
Leu424Arg (g.71075T > G)	Case 1: 30 year old Polish male. Five family members with EOD and myoclonic jerks, all died within 4-8 years of disease onset.	Progressive memory and language impairment started at the age of 30. Sporadic myoclonic jerks and mild left hemiparesis a year later.	Moderate general brain atrophy with enlargement of ventricles and subarachnoid spaces (MRI, CT); photopenic focus in left occipital lobe and lowered brain-cerebellum ratio (SPECT). Neuropathological examination not done.
	Case 2: 38 year old Dutch male. Mother with cognitive decline and gait difficulties at 35, died at 39 with severe dementia, epilepsy and hallucinations. Grandfather with similar symptoms died in mid-30s.	Initially: incipient memory problems, bradyphrenia, slurred speech, spastic and ataxic gate, MMSE score 24/30. Later: dementia, spastic paraparesis, frontal executive dysfunction mimicking familial CJD and FTD.	Bilateral posterior parietofrontal atrophy, no white matter abnormalities (MRI); CSF biomarkers positive for AD (↑p-tau and total tau, ↓ amyloid β1_42_ concentration). Neuropathological examination not done.
	Case 3: 34 years old Polish female with a positive family history for EOD, personality changes and involuntary movements. F++ather died at 37, his sister—at 40 with such symptoms.	Behavioral and personality changes (apathy and aggression) and cognitive decline (MMSE 18/30), severe involuntary movements—grimacing, smacking, and lips puckering, choreic movements in the upper and lower extremities, gait problems. Seizures occurred several months later.	Moderate general atrophy with ventricles and subarachnoid space enlargement (CT); Generalized diffuse slowing and a decrease in reactivity of the basic rhythm (EEG). Neuropathological examination not done.
Leu424His (g.71075T > A)	Case 1: 39 years old Polish female with unknown family history;	Symptoms at 39 years: depression, anxiety, personality changes and social inhibition. Soon after that—visual and auditory hallucinations and stereotyped behaviors; 1.5 year after that: deficit in short-term memory, attention and executive functions, MMSE 17/30, EPS (bradykinesia, rigidity) and bilateral primitive reflexes, rapid progression of symptoms.	Generalized cerebral atrophy (MRI); Diffuse cerebral hypoperfusion (SPECT). Neuropathological examination not done.
	Case 2: Three individuals from a French EOD family.	Onset of dementia symptoms between 38 and 42 years. Further details not provided.	N.A. Neuropathological examination not done.
Leu424Phe (g.71074C > T)	Two cases in a Bulgarian family with five other members affected by behavioral abnormalities and dementia.	Patient 1—depression at 54 years of age followed by dementia and grand mal epilepsy; Patient 2—memory loss, aggression, delusions, and hallucinations at age 60, followed by development of dementia.	Severe brain atrophy with white-matter changes. Neuropathological examination not done.
Leu424Val (g.71074C > G)	Case 1: 36 years old Spanish female with family history for late onset dementia (> 80 years) of maternal grandmother.	At 30: attentional deficit, forgetfulness and dysexecutive symptoms (difficulties driving etc.) At 34: irritability, despair, verbal aggression outbursts, impulsive behavior, sporadic suicide ideation, persisting memory impairment. At 35: aberrant behavior, bradylalia, dysnomia, mixed dysphasia, acalculia, ideatory apraxia, catatonic state, and ECT. Postural dystonia on neurological exam, followed by status epilepticus.	Generalized cortico-subcortical atrophy more pronounced in dorsal prefrontal, posterior parietal, and anterior temporal regions; hippocampus was proportional to the rest of the temporal lobe (CT and MRI). Diffuse slowing without other specific features (EEG). Second CT (17 months after the first): broadening of ventricles and Silvian fissures (i.e., progrssive atrophy); Extensive hypoperfusion affecting frontal, temporal and parietal lobes (SPECT). Neuropathological examination not done.
		At 36: stereotyped vocalizations and laughing, mydriatic hyporeflective pupils, grasping reflex, slight trunk flexion, urine incontinence, myoclonic jerks, several generalized motor seizures.	
	Case 2: 39 years old male whose father had rapidly progressing dementia started at 44 years with severe memory problems, dyspraxia, avolition (died in 1985 aged 48).	At 38: mild slowing of psychomotorics and responsivity. At 39: bradyphrenia, slurred speech, dysnomic signs, emotional ability with transient episodes of sadness and irritability, followed by visuospatial difficulties and progressive short-term memory and attention impairment (MMSE 14/30), dyspraxia and dysgraphia. Vertigo, tinnitus and horizontal nystagmus noted in neurological exam. At 40: global aphasia, acalculia, apraxia, extreme bradykinesia, mild hand tremor, Parkinsonian facial expression, ataxina, gait disturbances, episodic verbal aggression outbursts. At 40: severe hand tremor almost absent at rest, extremely expressed aphasia and visual agnosia, bed-ridden (dies at 41 from complicated respiratory infection).	Cortical atrophy predominantly in temporal and frontal cortex (CT and MRI). Neuropathological examination not done.
Leu424Pro (g.73685864T > C)	51-year-old male patient from Turkey whose uncle had dementia and died at appr. 40 years of age. Proband’s father has died from cancer aged 45 with preceding minor cognitive impairment.	Initial symptoms: deterioration of short-term memory and visuospatial abilities started at the age of 47. No significant neurological sings. MMSE score: 22/30.	Prominent medial temporal lobe atrophy and global cortical atrophy (MRI); Hypometabolism in parietal areas, the precuneus, and the posterior cingulate cortex (PET); Decreased amyloid level in CSF. Neuropathological examination not done.
		Currently followed-up, demonstrates benign course of the disease, no rapid progression.	

Drawing a comparison between our case and the one described by Robles et al. (25), brings several interesting facts to the fore. First and most interesting, as in Leu424Arg (g.71075T > G) and Leu424His (g.71075T > A) variants, an identical polymorphism is associated with two quite distinct phenotypic presentations—in the Spanish patient, initial attentional deficit, behavioral deviations, and executive dysfunction lasting several years were observed, followed by somewhat milder impairment of memory and speech, while prominent and papidly progressing primary disturbance in episodic memory and speech were initially seen in our patient subsequently supervened by marked parkinsonian symptoms. Second, there is a difference in neuroimaging findings: a predominantly frontotemporal cortical atrophy was observed in our case and diffuse cortico-subcortical atrophy was reported by Robles et al. (25). Third, seizures and myoclonus were present in the patient from Spain, while no incidence of such has been observed in our patient. Besides that, age of onset of cognitive symptoms in the Spanish patient was earlier—between 30 and 34 years vs. approx. 38–39 years in our case. What the two subjects have in common is the abrupt occurrence of quite similar symptoms of irritability, despair, and verbal outbursts prior to manifestation of severe cognitive decline. In addition, in both subjects gate disturbances were noted.

If we are to compare the phenotypic expression of our patient with the majority of other *PSEN-1* mutations reported in literature, the most important notion here is that this is not a “typical” *PSEN-1* polymorphism, i.e., such as presenting with spastic paraparesis. Another substantial distinction is the absence of myoclonus, while the extrapyramidal symptoms, ataxia and gait disturbances we observed are in line with other reports in literature (34, 40–42). So is the age of onset—shortly after mid-30s—which is in accordance with data with the other two mutations in the same codon in exon 12 of the gene—Leu424Arg (33–35) and Leu424His (36, 37) but in striking non-compliance with Leu424Phe and Leu424Pro variants associated with significantly later onset of symptoms (> 55 years and > 47 years, respectively) in the affected individuals (38, 39). The last two cases, supplemented by the fact that our patient’s father also did show later occurrence of symptoms (44 years) challenges the suggestion that age of AD onset is strongly determined by the position of the mutation of *PSEN-1* gene (43) but rather depend on intramolecular or intermolecular interactions of PSEN-1 with other proteins (39). At the same time, however, they support the observation of later occurrence of symptoms in mutations after codon 200 compared to those before it which has been stated in literature also as type 2 vs. type 1 pathology in EOAD (13). Besides, the purported modifying role of apoliprotein E4 status on age of onset of familial AD (44) is also questioned by the case subject who did not carry high risk E4 alelle and yet symptoms occurred five years earlier compared to his father. Clearly, much more identified and investigated EOAD pedigrees are needed in the future to confirm or refute these inferences.

Another point that should be emphasized is the co-occuring lupus erythematosus (LE) with predominant skin manifestations diagnosed in 2007. In our opinion, the presense of this conditon verified by an in-hospital histological examination several months prior to our first encounter is very interesting and suggests a possible pathogenic link between AD and LE *via* altered *PSEN-1* function. This assumption is supported by at least two lines of evidence. First, emerging data show that in addition to AD, γ-secretase is also involved in organ-specific (e.g., multiple sclerosis) as well as in generalized (e.g., systemic lupus erythematodes) autoimune diseases *via* the B-cell maturation antigen (BCMA) which is a part of the BAFF-APRIL receptor ligand system controlling activation and survival of B-lyphocytes (45). Second, γ-secretase operates several substrates including the Notch receptor protein (46) which is a part of Notch signalling pathway regulating cell proliferation but also implicated in innate immunity and inflamation, including rheumatioid arthritis and systemic lupus (47). Given the current lack of data supporting statistically significant co-occurrence of EOAD and LE, their combination in the presented case should be regarded as purely random. Yet, we consider screening of known *PSEN-1* mutation carriers for laboratory or clinical symptoms of autoimune diseases scientifically meaningful and recommend it to all researchers in the field.

**Figure 5:**
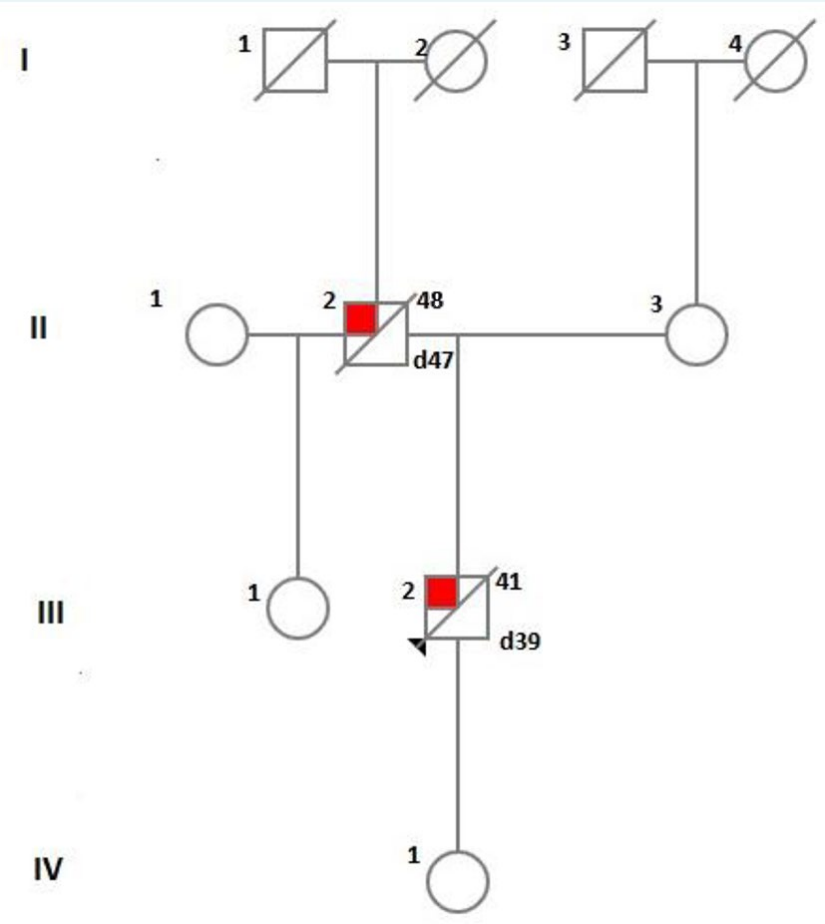
A diagram showing the family tree of the index patient.

A certain limitation of our exploration is the paucity of information concerning precise characteristics and chronology of the clinical manifestation of our patient’s father, as well as the lack of neuropathological examination of patient himself which would have probably revealed the characteristics and localization of amyloid plaques and amyloid angiopathy and the eventual presence of Lewy bodies. Besides, the subject’s 18 years old daughter who currently does not manifest any neuropsychiatric symptoms has not been genetically tested yet due to non-provision of consent. Furthermore, the patient’s sister (aged 45) from the first marriage of his father (see family tree on **Figure 5**) could not be found and examined and the last information for her showing absence of cognitive impairment dates back from several years ago.

What underlies phenotypic heterogeneity in *PSEN-1* mutations, once again confirmed by the presented case, remains to be clarified. A number of factors combining to modulate pathogenic polymorphisms have been discussed in literature, for example interaction between mutated PS-1 and its protein partners in γ-secretase (48), brain ischaemia (49), D-amino acid oxidase activator (DAOA) an other genes (50), education (44), racial and ethnic background (24). Obviously, future genetic, molecular, and bioinformatics studies are needed to address this issue.

## Conclusion

In addition to further supporting the vast phenotypic variability produced by genetically identical missense mutations in the *PSEN-1* gene, our study confirms that in EOAD with positive family history, WES can be especially informative. In our view, it should be considered as a first-line examination given the ease of genome-wide assessment, the high diagnostic value, the accuracy of mutation detection, and the relatively affordable price of DNA extraction. Easily accessible online bioinformatics tools now simplify and expedite data review relevant to symptoms. Genetic mutations in AD have large prognostic value and, possibly, treatment implications in the future. Moreover, with informed consent provided, they enable longitudinal re-examination and new findings.

## Data Availability Statement

All datasets generated for this study are included in the article/supplementary material.

## Ethics Statement

The studies involving human participants were reviewed and approved by Ethics committee of Medical University Pleven, Bulgaria. The patients/participants provided their written informed consent to participate in this study. Written informed consent was obtained from the individual(s) for the publication of any potentially identifiable images or data included in this article.

## Author Contributions

KS: initial and subsequent patient psychiatric examination, obtaining and sending patient DNA sample, literature review and manuscript preparation. MSt: subsequent (follow-up) psychiatric examination, manuscript preparation. PC: subsequent (follow-up) psychiatric examination, manuscript preparation. LI: initial and subsequent neurological examination. MSw: literature review, manuscript preparation. The publication has been supported by a research grant from Medical University Pleven, Bulgaria.

## Funding

This publication has been supported by a research grant from Medical University Pleven, Bulgaria.

## Conflict of Interest

The authors declare that the research was conducted in the absence of any commercial or financial relationships that could be construed as a potential conflict of interest.

## References

[1] World Health organization Global action plan on the public health response to dementia 2017–2025. (2017). Geneva: World Health Organization, Licence: CC BY-NC-SA 3.0 IGO. Geneva, Switzerland https://apps.who.int/iris/bitstream/handle/10665/259615/9789241513487-eng.pdf?sequence=1.

[2] Ryan J Fransquet P Wrigglesworth J Lacaze P Phenotypic heterogeneity in dementia: a challenge for epidemiology and biomarker studies. Front Public Health (2018) 6:181. 10.3389/fpubh.2018.00181 29971228 PMC6018385

[3] Beach TG Monsell SE Phillips LE Kukull W Accuracy of the clinical diagnosis of Alzheimer disease at National Institute on Aging Alzheimer Disease Centers, 2005-2010 . J Neuropathol Exp Neurol (2012) 71(4):266–73. 10.1097/NEN.0b013e31824b211b PMC3331862 22437338

[4] Koriath C Kenny J Adamson G Druyeh R Taylor W Beck J Predictors for a dementia gene mutation based on gene-panel next-generation sequencing of a large dementia referral series. Mol Psychiatry (2018). 10.1038/s41380-018-0224-0 PMC6330090 30279455

[5] Paulson HL Igo I Genetics of dementia. Semin Neurol (2011) 31(5):449–60. 10.1055/s-0031-1299784 PMC3545709 22266883

[6] Loy CT Schofield PR Turner AM Kwok JBJ Genetics of dementia. Lancet (2014) 383(9919):828–40. 10.1016/S0140-6736(13)60630-3 23927914

[7] Vieira RT Caixeta L Machado S Silva AC Nardi AE Arias-Carrión O Epidemiology of early-onset dementia: a review of the literature. Clin Pract Epidemiol Ment Health (2013) 9:88–95. 10.2174/1745017901309010088 23878613 PMC3715758

[8] Perrone F Cacace R Van Mossevelde S Van den Bossche T De Deyn P Gras P Genetic screening in early onset dementia with unclear phenotype: relevance of clinical diagnosis. Neurobiol Aging (2018) 69:292.e7–292.e14. 10.1016/j.neurobiolaging.2018.04.015 29859640

[9] Zalar B Maver A Kovanda A Peterlin A Peterlin B Clinical exome sequencing in dementias: a preliminary study. Psychiatr Danubina (2018) 30(2):216–9. 10.24869/psyd.2018.216 29930232

[10] Zhu XC Tan L Wang HF Jiang T Cao L Wang C Rate of early onset Alzheimer’s disease: a systematic review and meta-analysis. Ann Transl Med (2015) 3(3):38. 10.3978/j.issn.2305-5839.2015.01.19 25815299 PMC4356853

[11] Munshi A Ahuja YR Genes associated with Alzheimer’s disease. Neurol Asia (2010) 15(2):109–18.

[12] Mendez MF The accurate diagnosis of early-onset dementia. Int J Psychiatry Med (2006) 36(4):401–12. 10.2190/Q6J4-R143-P630-KW41 17407994

[13] Ryan NS Rossor MN Correlating familial Alzheimer’s disease gene mutations with clinical phenotype. Biomark Med (2010) 4(1):99–112. 10.2217/bmm.09.92 20387306 PMC3937872

[14] Livingston G Sommerland A Ortega V Costafreda S Dementia prevention, intervention and care. Lancet (2017) 390(10113):2673–734. 10.1016/S0140-6736(17)31363-6 28735855

[15] Giri M Zhang M Lü Y Genes associated with Alzheimer’s disease: an overview and current status. Clin Interv Aging (2016) 11:665–81. 10.2147/CIA.S105769 PMC4876682 27274215

[16] Janssen JC Beck JA Campbell TA Dickinson A Fox NC Harvey RJ Early onset familial Alzheimer’s disease: Mutation frequency in 31 families. Neurol (2003) 60(2):235–9. 10.1212/01.wnl.0000042088.22694.e3 12552037

[17] Lanoiselée H-M Nicolas G Wallon D Rovelet-Lecrux A Lacour M Rousseau S APP, PSEN1, and PSEN2mutations in early-onset Alzheimer disease: A genetic screening study of familial and sporadiccases. PloSMed (2017) 14(3):e1002270. 10.1371/journal.pmed.1002270 PMC5370101 28350801

[18] Alzheimer Disease and Frontotemporal Dementia Mutation Database (0000) Available online at: http://www.molgen.ua.ac.be/ADMutations.

[19] Hardy JA Higgins GA Alzheimer’s disease: the amyloid cascade hypothesis. Science (1992) 256(5054):184–5. 10.1126/science.1566067 1566067

[20] Karlstrom H Brooks WS Kwok JB Broe GA Kril JJ McCann H Variable phenotype of Alzheimer’s disease with spastic paraparesis. J Neurochem (2008) 104(3):573–83. 10.1111/j.1471-4159.2007.05038.x. 17995932

[21] Roks G Van Harskamp F De Koning I Cruts M De Jonghe C Kumar-Singh S Presentation of amyloidosis in carriers of the codon 692 mutation in the amyloid precursor protein gene (APP692). Brain (2000) 123(10):2130–40. 10.1093/brain/123.10.2130 11004129

[22] Jayadev S Leverenz JB Steinbart E Stahl J Klunk W Yu CE Alzheimer’s disease phenotypes and genotypes associated with mutations in presenilin 2. Brain (2010) 133(4):1143–54. 10.1093/brain/awq033 PMC2850581 20375137

[23] Delgado-Morales R Esteller M Opening up the DNA methylome of dementia. Mol Psychiatry (2017) 22:485–96. 10.1038/mp.2016.242 PMC5378809 28044062

[24] Larner AJ Doran M Genotype-phenotype relationships of presenilin-1 mutations in Alzheimer’s disease: an update. J Alzheimers Dis (2009) 17(2):259–65. 10.3233/JAD-2009-1042 19221408

[25] Robles A Sobrido MJ García-Murias M Prieto JM Lema M Santos D Clinical Picture of a Patient With a Novel PSEN1 Mutation (L424V). Am J Alzheimer’s Dis Other Dem (2009) 24(1):40–5. 10.1177/1533317508324272 PMC10846114 19001354

[26] Chau DM Crump CJ Villa JC Scheinberg DA Li YM Familial Alzheimer disease presenilin-1 mutations alter the active site conformation of γ-secretase. J Biol Chem (2012) 287(21):17288–96. 10.1074/jbc.M111.300483 PMC3366784 22461631

[27] Bettens K Sleegers K Van Broeckhoven C Genetic insights in Alzheimer’s disease. Lancet Neurol (2013) 12(1):92–104. 23237904 10.1016/S1474-4422(12)70259-4

[28] Tanzi R The genetics of Alzheimer disease. Cold Spring Harb Perspect Med (2012) 2(10):a006296. 10.1101/cshperspect.a006296 23028126 PMC3475404

[29] Sun L Zhou R Yang G Shi Y Analysis of 138 pathogenic mutations in presenilin-1 on the *in vitro* production of Aβ42 and Aβ40 peptides by γ-secretase. Proc Natl Acad Sci USA (2017) 114(4):E476–85. 10.1073/pnas.1618657114 PMC5278480 27930341

[30] Ryman DC Acosta-Baena N Aisen PS Bird T Danek A Fox NC Symptom onset in autosomal dominant Alzheimer disease: a systematic review and meta-analysis. Neurol (2014) 83(3):253–60. 10.1212/WNL.0000000000000596 PMC4117367 24928124

[31] Bagyinszky E Youn YC An SS Kim S The genetics of Alzheimer disease. Clin Interv Aging (2014) 9:5359551. 10.2147/CIA.S51571 PMC3979693 24729694

[32] Kowalska A Forsell C Florczak J Pruchnik-Wolińska D Modestowicz R Paprzycki W A Polish pedigree with Alzheimer’s disease determined by a novel mutation in exon 12 of the presenilin 1 gene: clinical and molecular characterization. Folia Neuropathol (1999) 37(1):57–61. 10337065

[33] Kowalska A Wender M Florczak J Pruchnik-Wolinska D Modestowicz R Szczech J Molecular genetics of Alzheimer’s disease: presenilin 1 gene analysis in a cohort of patients from the Poznań region. J Appl Genet (2003) 44(2):231–4. 12817569

[34] De Bot ST Kremer HP Dooijes D Verbeek MM CSF studies facilitate DNA diagnosis in familial Alzheimer’s disease due to a presenilin-1 mutation. J Alzheimer’s Dis (2009) 17(1):53–7. 10.3233/JAD-2009-1038 19494431

[35] Klimkowicz-Mrowiez A Bodzioh M Szszudlik A Slowik A Clinical presentation of early-onset Alzheimer’s disease as a result of mutation in exon 12 of the PSEN-I gene. Am J Alzheimer’s Dis Other Dem (2014) 29(8):732–4. 10.1177/1533317514536599 PMC10852588 24906965

[36] Golan M Lipczynska-Lojkowska W Krzysko KA Styczynska M Luczywek E Filipek S Two novel mutations in presenilin 1 (PSEN1) gene connected with atypical familial early-onset Alzheimer’s disease (EOAD). Alzheimer’s and Parkinson’s Diseases: Insights, Progress and Perspectives. 7th International Conference AD/PD; 2005 Mar 9-13; Sorrento, Italy Book of Abstracts: p. 24.

[37] Raux G Guyant-Marechal L Martin C Bou J Penet C Brice A Molecular diagnosis of autosomal dominant early onset Alzheimer’s disease: an update. J Med Genet (2005) 42(10):793–5. 10.1136/jmg.2005.033456 PMC1735922 16033913

[38] Mehrabian S Traykov LT Jordanova A Rademakers R Cruts M Raycheva MR Novel PSEN1 gene mutation in a large Bulgarian pedigree with Alzheimer’s disease and atypical phenotype. Eur J Neurol (2006) 13(Suppl. 2):41.

[39] Guven G Erginel-Unaltuna N Samanci B Gulec C Hanagasi H Bilgic B A patient with early-onset Alzheimer’s disease with a novel PSEN1 p.Leu424Pro mutation. Neurobiol Aging (2019). 10.1016/j.neurobiolaging.2019.05.014 pii: . 31296348

[40] Ikeda M Yonemura K Yoshida J Fujita Y Hashimoto Y Ishiguro K Diverse neurological symptoms presenting severe dementia, psychiatric symptoms and motor deficits in early-onset familial Alzhemer’s disease. Alzheimer Dement (2008) 4(suppl 2):T522. /10.1016/j.jalz.2008.05.1582

[41] Ikeda M Yonemura K Harigaya Y Okamoto K Mutant presenilin-1 M233L presenting severe parkinsonism and common patterning of temporal processes in neurological symptoms. Alzheimer Dement (2006) 2(suppl 1):S255.

[42] Appel-Cresswell S Guella I Lehman A Foti D Farrer MJ *PSEN1* p.Met233Val in a complex neurodegenerative movement and neuropsychiatric disorder. J Mov Disord (2018) 11(1):45–8. 10.14802/jmd.17066 PMC5790629 29316780

[43] Cruts M Van Broeckhoven C Presenilin mutations in Alzheimer’s disease. Hum Mutat (1998) 11(3):183–90. 10.1002/(SICI)1098-1004(1998)11:3<183::AID-HUMU1>3.0.CO;2-J 9521418

[44] Pastor P Roe CM Villegas A Bedoya G Chakraverty S Garcia G Apolipoprotein E epsilon 4 modifies Alzheimer’s disease onset in an E280A PS1 kindred. Ann Neurol (2003) 54(2):163–9. 10.1002/ana.10636 12891668

[45] Laurent SA Hoffmann FS Kuhn PH Cheng Q Chu Y Schmidt-Supprian M γ-Secretase directly sheds the survival receptor BCMA from plasma cells. Nat Commun (2015) 6:7333. 10.1038/ncomms8333 26065893 PMC4490565

[46] De Strooper B Iwatsubo T Wolfe MS Presenilins and γ-secretase: structure, function, and role in Alzheimer Disease. Cold Spring Harb Perspect Med (2012) 2(1):a006304. 10.1101/cshperspect.a006304 22315713 PMC3253024

[47] Shang Y Smith S Hu X Role of Notch signaling in regulating innate immunity and inflammation in health and disease. Protein Cell (2016) 7(3):159–74. 10.1007/s13238-016-0250-0 PMC4791423 26936847

[48] Zekanowski C Golan MP Krzyśko KA Lipczyńska-Łojkowska W Filipek S Kowalska A Two novel presenilin 1 gene mutations connected with frontotemporal dementia-like clinical phenotype: genetic and bioinformatic assessment. Exp Neurol (2006) 200(1):82–8. 10.1016/j.expneurol.2006.01.022 16546171

[49] Kocki J Ułamek-Kozioł M Bogucka-Kocka A Januszewski S Jabłonski M Gil-Kulik P Dysregulation of amyloid-β protein precursor, γ-secretase, presenilin 1 and 2 genes in the rat selectively vulnerable CA1 subﬁeld of hippocampus following transient global brain ischemia. J Alzheimer’s Dis (2015) 47(4):1047–56. 10.3233/JAD-150299 PMC4923727 26401782

[50] Vélez JI Rivera D Mastronardi CA Patel HR Tobón C Villegas A (2016). A mutation in DAOA modifies the age of onset in PSEN1 E280A Alzheimer’s disease. Neural Plast 2016(9760314):7. 10.1155/2016/9760314 PMC4753688 26949549

